# Comparison of two survey methods for estimating unplanned pregnancy, Bangladesh

**DOI:** 10.2471/BLT.23.290262

**Published:** 2024-05-21

**Authors:** Md Nuruzzaman Khan, Shimlin Jahan Khanam, Melissa L Harris

**Affiliations:** a Department of Population Science, Jatiya Kabi Nazrul Islam University, Trishal-2204, Mymensingh, Bangladesh.; b Centre for Women’s Health Research, University of Newcastle, Callaghan, Australia.

## Abstract

**Objective:**

To compare the prevalence of unintended pregnancy measured by the Demographic and Health Survey (DHS) and the London Measure of Unplanned Pregnancy in Bangladesh, and explore the extent of discordance between the measures and the factors associated with the discordance.

**Methods:**

In 2023, we conducted a cross-sectional survey in four randomly selected districts in Bangladesh: Kurigram, Mymensingh, Pabna and Satkhira. We randomly selected 20 hospitals, five from each district. We collected data from 1200 women who had recently delivered a baby and were visiting the hospitals for postnatal care. We interviewed the women about their pregnancy intention in their last pregnancy using questions in the DHS and the London Measure of Unplanned Pregnancy and examined the discordance in their responses. We used multivariable logistic regression analysis to identify factors associated with discordant responses in reported pregnancy intention.

**Findings:**

The prevalence of unintended pregnancy was 24.3% (292/1200) using the DHS measure and 31.0% (373/1200) using the London Measure of Unplanned Pregnancy. Discordance in responses to pregnancy intention between the two measures was 27.1% (325/1200). Factors associated with discordance were older age, female sex of the last child born, having more than two children, being in a poorer wealth quintile, living in a rural area and living in Kurigram district.

**Conclusion:**

The prevalence of unintended pregnancy in Bangladesh measured by the DHS measure may be an underestimate, suggesting that the adverse effects of unintended pregnancy are greater than realized and emphasizing the need to bolster Bangladesh’s family planning programme.

## Résumé

**Objectif:**

Comparer la prévalence des grossesses non désirées mesurée par l’Enquête démographique et de santé (EDS) et par la London Measure of Unplanned Pregnancy au Bangladesh et explorer l’étendue de la discordance entre les mesures et les facteurs associés à cette discordance.

**Méthodes:**

En 2023, nous avons mené une enquête transversale dans quatre districts du Bangladesh choisis au hasard: Kurigram, Mymensingh, Pabna et Satkhira. Nous avons choisi au hasard 20 hôpitaux, cinq par district. Nous avons recueilli des données auprès de 1200 femmes qui avaient récemment accouché et qui se rendaient à l’hôpital pour des soins postnatals. Nous avons interrogé ces femmes sur le désir de leur dernière grossesse en leur posant des questions issues de l’Enquête démographique et de santé et de la London Measure of Unplanned Pregnancy. Nous avons ensuite examiné la discordance entre leurs réponses. Nous avons appliqué une analyse de régression logistique multivariable afin d’identifier les facteurs associés aux réponses discordantes dans les intentions de grossesse déclarées.

**Résultats:**

La prévalence des grossesses non désirées était de 24,3% (292/1200) selon l’Enquête démographique et de santé et de 31,0% (373/1200) selon la London Measure of Unplanned Pregnancy. La discordance dans les réponses portant sur l’intention de grossesse entre les deux mesures était de 27,1% (325/1200). Les facteurs associés à la discordance étaient l’âge avancé, le sexe féminin du dernier enfant né, le fait d’avoir plus de deux enfants, d’appartenir à un quintile de richesse inférieur, de vivre dans une zone rurale et de vivre dans le district de Kurigram.

**Conclusion:**

Il se peut que la prévalence des grossesses non désirées au Bangladesh, telle que mesurée par l’Enquête démographique et de santé, soit sous-estimée, ce qui suggère que les effets défavorables des grossesses non désirées sont plus importants qu’on ne le pense et souligne la nécessité de renforcer le programme de planification familiale du Bangladesh.

## Resumen

**Objetivo:**

Comparar la prevalencia del embarazo no deseado medida por la Encuesta Demográfica y de Salud (EDS) y la London Measure of Unplanned Pregnancy en Bangladesh, y explorar el grado de discordancia entre las medidas y los factores asociados con la discordancia.

**Métodos:**

En 2023, se realizó una encuesta transversal en cuatro distritos de Bangladesh seleccionados al azar: Kurigram, Mymensingh, Pabna y Satkhira. Se seleccionaron al azar 20 hospitales, cinco de cada distrito. Se recopilaron datos de 1200 mujeres que habían dado a luz recientemente y acudían a los hospitales para recibir atención posnatal. Se entrevistó a las mujeres sobre su intención de embarazo en su último embarazo utilizando preguntas de la EDS y la London Measure of Unplanned Pregnancy, y se examinó la discordancia en sus respuestas. Se utilizó un análisis de regresión logística multivariable para identificar los factores asociados a las respuestas discordantes en la intención de embarazo declarada.

**Resultados:**

La prevalencia de embarazos no deseados fue del 24,3% (292/1200) utilizando la medida EDS y del 31,0% (373/1200) utilizando la London Measure of Unplanned Pregnancy. La discordancia en las respuestas a la intención de embarazo entre las dos medidas fue del 27,1% (325/1200). Los factores asociados a la discordancia fueron la edad avanzada, el sexo femenino del último hijo nacido, tener más de dos hijos, pertenecer a un quintil de riqueza más pobre, vivir en un área rural y vivir en el distrito de Kurigram.

**Conclusión:**

La prevalencia de embarazos no deseados en Bangladesh medida por la EDS puede ser una subestimación, lo que sugiere que los efectos adversos de los embarazos no deseados son mayores de lo que se cree y subraya la necesidad de reforzar el programa de planificación familiar de Bangladesh.

## ملخص

**الغرض:**

إجراء مقارنة بين انتشار الحمل غير المقصود الذي تم قياسه بواسطة المسح السكاني والصحي (DHS)، والانتشار الذي تم قياسه باستخدام مقياس لندن للحمل غير المخطط له (London Measure of Unplanned Pregnancy) في بنغلاديش، إلى جانب استكشاف مدى التعارض بين المقياسين، والعوامل المرتبطة بهذا التعارض.

**الطريقة:**

في عام 2023، قمنا بإجراء مسح متعدد القطاعات في أربع مناطق تم اختيارها عشوائيًا في بنغلاديش وهي: كوريجرام (Kurigram)، وميمينسينغ (Mymensingh)، وبابنا (Pabna)، وساتخيرا (Satkhira). قمنا باختيار 20 مستشفى بشكل عشوائي، خمسة من كل منطقة. وقمنا بجمع البيانات من 1200 سيدة أنجبن أطفالاً مؤخرًا، وكن في زيارة للمستشفى للحصول على رعاية ما بعد الولادة. وأجرينا مقابلات مع السيدات بخصوص نيتهن لحدوث الحمل في حملهن الأخير، وذلك باستخدام أسئلة من كل من المسح السكاني والصحي، ومقياس لندن للحمل غير المخطط له، وفحصنا التعارض في إجاباتهن. استخدمنا تحليل التحوف اللوجستي متعدد المتغيرات لتحديد العوامل المرتبطة بالإجابات المتعارضة في نية الحمل المبلغ عنها.

**النتائج:**

كان معدل انتشار الحمل غير المقصود هو %24.3 (292/1200)، وذلك باستخدام مقياس المسح السكاني والصحي، بينما كان %31.0 (373/1200) باستخدام مقياس لندن للحمل غير المخطط له. كان التعارض في الإجابات لنية الحمل بين المقياسين هو %27.1 (325/1200). كانت العوامل المرتبطة بالتعارض هي العمر الأكبر، وأن آخر طفل مولود كان أنثى، وإنجاب أكثر من طفلين، والتواجد في فئة خُمسية أكثر فقرًا، والحياة في منطقة ريفية، والحياة في منطقة كوريجرام.

**الاستنتاج:**

إن معدل انتشار الحمل غير المقصود فيه في بنغلاديش الذي تم قياسه بواسطة مقياس المسح السكاني والصحي قد يكون تقديريًا أقل من الواقع، مما يشير إلى أن التأثيرات السلبية للحمل غير المقصود هي أكبر من المتوقعة، ويؤكد الحاجة إلى النهوض ببرنامج تنظيم الأسرة في بنغلاديش.

## 摘要

**目的:**

比较在孟加拉国通过人口统计与健康调查 (DHS) 和伦敦意外妊娠量表测量的意外怀孕发生率，并探讨不同测量方法所获得结果的不一致程度以及导致不一致的因素。

**方法:**

2023 年，我们在孟加拉国随机选择的四个地区进行了横断面调查：古里格拉姆县、迈门辛、帕布纳和萨德基拉。我们随机选择了 20 家医院，每个区 5 家。我们收集了 1,200 名最近生过孩子并在医院接受产后护理的妇女的相关数据。我们使用 DHS 和伦敦意外妊娠量表中的问题采访了这些妇女，以了解她们上次怀孕时的妊娠意愿，并核实了她们回答中不一致的地方。我们使用多变量逻辑回归分析来确定导致与报告中对妊娠意愿回答不一致的因素。

**结果:**

通过 DHS 测量的意外怀孕发生率为 24.3% (292/1200)，而通过伦敦意外妊娠量表测量的意外怀孕发生率为 31.0% (373/1200)。两种方法所得到妊娠意愿回答的不一致率为 27.1% (325/1200)。导致结果不一致的因素包括年龄较大、最后出生的是女孩、有不止两个孩子、家庭收入水平属于较贫困阶层、居住在农村地区和居住在古里格拉姆县地区。

**结论:**

根据 DHS 测量结果来看，孟加拉国意外怀孕的发生率可能被低估了，这表明意外怀孕的不利影响比人们意识到的要大，同时突出了孟加拉国需要完善计划生育项目的必要性。

## Резюме

**Цель:**

Сравнить распространенность случаев незапланированной беременности, измеренную с помощью демографических и медико-санитарных обследований (Demographic and Health Survey, DHS) и Лондонского показателя незапланированной беременности в Бангладеш, а также изучить степень расхождения между этими показателями и факторы, связанные с этим расхождением.

**Методы:**

В 2023 году было проведено перекрестное исследование в четырех случайно выбранных районах Бангладеш: Куриграм, Майменсингх, Пабна и Сатхира. Случайным образом было отобрано 20 больниц, по пять из каждого района. Были собраны данные 1200 женщин, которые недавно родили ребенка и посещали больницы для послеродового наблюдения. По результатам опроса женщин о намерении забеременеть во время последней беременности при помощи вопросов из анкет DHS и Лондонского показателя незапланированной беременности были выявлены расхождения в их ответах. Для выявления факторов, связанных с разногласиями в ответах о намерении забеременеть, использовался многофакторный логистический регрессионный анализ.

**Результаты:**

Распространенность незапланированной беременности составила 24,3% (292/1200) по показателю DHS и 31,0% (373/1200) по Лондонскому показателю незапланированной беременности. Расхождения в ответах на вопрос о намерении забеременеть между двумя показателями составили 27,1% (325/1200). В числе факторов, связанных с несоответствием, были: более старший возраст матерей, женский пол последнего рожденного ребенка, наличие более двух детей, принадлежность к более бедному квинтилю благосостояния, проживание в сельской местности и проживание в районе Куриграм.

**Вывод:**

Уровень распространенности нежелательной беременности в Бангладеш, определенный по данным DHS, может быть занижен. Это свидетельствует о том, что негативные последствия нежелательной беременности более значительны, чем предполагается, и подчеркивает необходимость усиления программы планирования семьи в Бангладеш.

## Introduction

Globally, about half of pregnancies (roughly 121 million) are unintended, with low- and middle-income countries bearing the brunt due to lower use of modern contraception and higher unmet contraceptive needs.^1^^,^^2^ In Bangladesh, unintended pregnancy is an important issue. In one study, 25.2% (1131/4493) of women with live births reported that the pregnancy was unintended.^3^ Unintended pregnancies can have substantial adverse effects on maternal and child health, especially in low- and middle-income countries such as Bangladesh, where safe abortion services are limited by law.^1^^,^^4^ Around two thirds of unintended pregnancies in low- and middle-income countries and Bangladesh result in induced abortions. These abortions account for an estimated 13.0% of total maternal deaths and incur substantial costs for treating abortion-related complications.^1^^,^^5^ Unintended pregnancies leading to live births can result in delayed or inaccessible maternal and child care, increasing the risk of adverse outcomes such as stillbirths, preterm births, low birth weight, child undernutrition, and neonatal and maternal mortality.^3^^–^^7^ Intergenerational effects of unintended pregnancy, including higher rates of school dropout, have also been observed in studies conducted in low- and middle-income countries such as Bangladesh.^8^^–^^10^ Increasing access to modern contraception and comprehensive sex education is vital to reduce unintended pregnancies in Bangladesh.^3^

The accuracy of current estimates of unintended pregnancy is a global concern, particularly in low- and middle-income countries where prospective data on reproductive events (e.g. conception and birth) are scarce, and data on unintended pregnancy usually come from nationally representative Demographic and Health Surveys (DHS).^5^ These surveys collect timing-based data on pregnancy intention using two questions. These questions have several limitations, including recall bias, particularly in accurately remembering pregnancy intentions at conception, and a higher likelihood of ambivalent responses, especially when the child’s sex differs from the sex the parents desire or when pregnancy intentions change after childbirth.^5^ The main reasons for these limitations include: (i) exclusion of women who have had induced abortions; (ii) lack of inclusion of partners’ perspectives, despite their important role in pregnancy decisions; and (iii) failure to consider contraceptive use or non-use status at conception.^11^^,^^12^ These factors can lead to ambivalence, denial and confusion about pregnancy, and misreporting of partners’ intentions, as a considerable portion of reported unintended pregnancies in low- and middle-income countries are wanted by the husband.^5^^,^^11^^,^^12^

To comprehensively measure pregnancy intention, multiple dimensions need to be considered, such as contraception use, feelings about pregnancy at conception and after birth, and partner agreement.^12^ The London Measure of Unplanned Pregnancy addresses these issues and can overcome the limitations of the DHS measure of pregnancy intention.^13^ However, the London Measure of Unplanned Pregnancy is not commonly used in low- and middle-income countries and it has not been integrated into national surveys.^11^^–^^14^ Furthermore, no previous investigation has been done into the discordance between the DHS measure of pregnancy intention and the London Measure of Unplanned Pregnancy or the factors contributing to this discordance. The aim of our study therefore was to estimate the prevalence of unintended pregnancy in Bangladesh using these two measures: the DHS timing-based estimate and the London Measure of Unplanned Pregnancy. We also sought to determine the degree of discordance between the prevalence estimates obtained from these two measures, and identify the factors that contribute to this discordance.

## Methods

### Study setting and sample

**Fig. 1:**
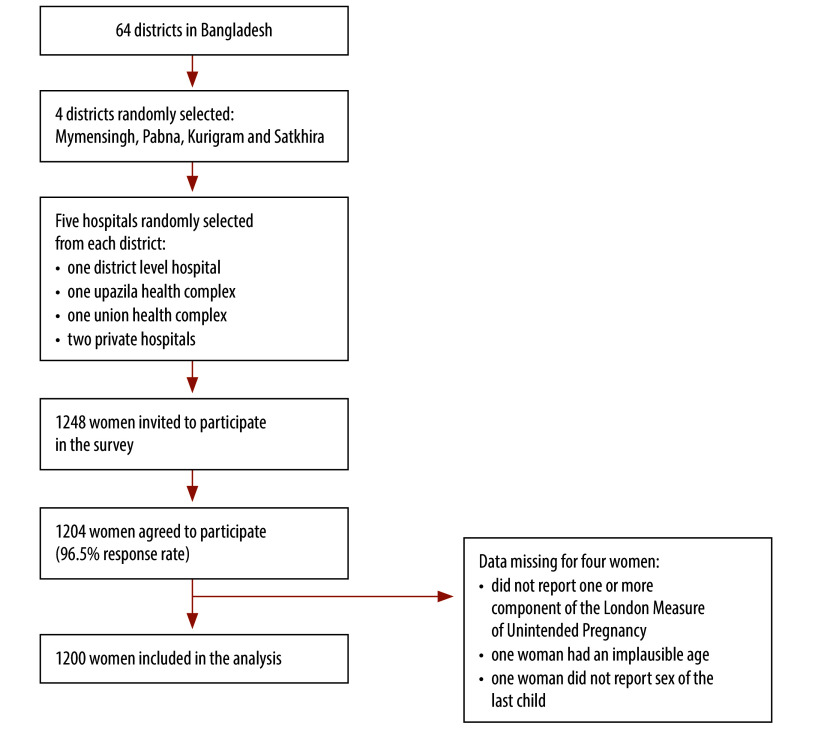
Flowchart of selection of women to interview for the comparison of survey methods for estimating unplanned pregnancy, Bangladesh, 2023

We used a cross-sectional survey design with a two-stage stratified random sampling method. First, using the lottery method, we randomly selected four districts in Bangladesh: Kurigram, Mymensingh, Pabna and Satkhira, from the 64 districts in total (Fig. 1). Then, we selected 20 hospitals, five from each district. We selected the one district-level hospital in each district. Then, in each district, we randomly chose one *upazila* (a district subunit) health complex from all *upazila* complexes; one union (smallest administrative unit) health complex from all union health complexes; and two private hospitals from all private hospitals. We obtained the list of hospitals providing maternal health-care services in the districts from the Ministry of Health and Family Welfare in Bangladesh. We used these hospitals as the sampling frame from which we selected eligible women. The eligibility criteria were women who had recently delivered a baby (either by caesarean section or vaginal delivery) and were visiting the selected hospitals for postnatal health-care services. Women who did not meet these criteria were excluded. Women were approached by the data collectors stationed at the hospitals.

We determined the sample size using the formula *n* = (*z*^2^ × *pq*)/*e*^2^, where *z* = 1.96 at the 95% confidence level, *p* is the sample proportion, *q* = 1 – *p* and *e* is the margin of error. We estimated a 10% non-response rate (114 women).^15^ We increased the sample size required (*n* = 378) by three times to improve the precision of the study, and added the estimated number of non-responders for a final sample size of 1248 women. 

### Data collection

We collected data from 12 January to 4 February 2023, through face-to-face interviews administered by trained data collectors holding bachelor or postgraduate degrees in population science and anthropology. We developed and tested a structured questionnaire which included the questions related to unintended pregnancy in both the DHS and the London Measure of Unplanned Pregnancy.^16^ We also incorporated other relevant questions from the Bangladesh DHS, such as respondents’ sociodemographic background. The questionnaire underwent field-testing, with subsequent refinements made based on feedback received during pre-testing from the respondents. 

### Ethical considerations

The Human Research Ethics Committee of Jatiya Kabi Kazi Nazrul Islam University approved the final version of the questionnaire and the data collection method (approval number: JKKNIU2023–07).

We obtained written or verbal informed consent from each respondent before data collection began. We informed the women of their right to withdraw at any time during the survey or to decline to answer any questions they felt uncomfortable with. Additionally, we assured the women of the confidentiality of their data which would not be shared with anyone in an identifiable form.

### Outcome measure

Our primary outcome was discordant responses in pregnancy intention (yes, no) between the DHS measure and London Measure of Unplanned Pregnancy. Both approaches are globally validated, including in Bangladesh, with the London Measure of Unplanned Pregnancy being considered the most up-to-date and comprehensive measure.^13^^,^^16^ We generated the discordant response variable by comparing estimates of pregnancy intention from both measures. In the DHS measure, eligible women (those with at least one live birth within 5 years of the survey) are asked two questions. The first question asks whether the woman intended to become pregnant at conception, with response options of yes or no. A negative response leads to a follow-up question to determine whether not wanting to be pregnant was temporary (i.e. she wanted to be pregnant at a later time) or permanent (i.e. she did not want any (more) children). We categorized responses into intended pregnancy (positive response to the first question) and unintended pregnancy (negative response to the first question, and later or not at all in the second question; online repository).^17^ The London Measure of Unplanned Pregnancy records pregnancy intention through six questions in relation to the woman’s last pregnancy: contraception use; attitude to timing of pregnancy; pregnancy intention; desire for a baby; discussion with partner about pregnancy; and actions taken to improve health in preparation for pregnancy (online repository).^17^ Based on responses and scores, this measure estimates unplanned pregnancy (score: 0–3), ambivalence to pregnancy (score: 4–9) and planned pregnancy (score: 10–12; online repository).^17^ We considered responses concordant if women reported similar pregnancy intentions in both measures (i.e. pregnancy rated intended and planned, or unintended and unplanned by both measures). Discordant responses were mismatches between the two measures (e.g. pregnancy rated intended by one measure and unintended by the other measure).

### Explanatory variables

We first compiled a list of potential explanatory variables from a literature review in low- and middle-income countries, particularly in Bangladesh. We considered research exploring determinants of unintended pregnancy measured through the DHS measure.^3^^,^^5^^,^^12^^–^^14^^,^^18^^,^^19^ Next, we checked that all these variables were included in our survey. Thus, the following explanatory variables were included: women’s age (≤ 19 years, 20–34 years or ≥ 35 years); education (no formal education, primary school, secondary school or higher education); formal employment (yes or no); number of children ever born (1–2 or > 2); partner’s education (no formal education, primary school, secondary school or higher education); partner’s occupation (agricultural worker, physical labourer, office worker, self-employed businessman or other); sex of last child (male or female); household wealth quintile (poorest, poorer, middle, richer or richest); place of residence (urban or rural); and district of residence (Kurigram, Mymensingh, Pabna and Satkhira).

### Statistical analysis

We used descriptive statistics to present baseline characteristics and cross-tabulation to show discordant pregnancy intention. We used multivariable logistic regression analysis to explore factors associated with discordance and report odds ratios (ORs) with corresponding 95% confidence intervals (CIs). We estimated crude and adjusted associations and accounted for multicollinearity before modelling. We used Stata, version 15.2 (StataCorp. LP, College Station, United States of America) for all analyses.

## Results

Of the 1248 women approached, 1204 agreed to participate in the survey, a response rate of 96.5%, which is consistent with other nationally representative health-related surveys in Bangladesh.^5^ We excluded four respondents due to missing data on key variables (Fig. 1). Thus, our analysis included data from 1200 respondents, and their background characteristics are summarized in Table 1. Of the 1200 respondents, 962 (80.2%) were aged 20–34 years; 480 (40.0%) had a secondary education; and 828 (69.0%) were not formally employed. As regards partners, 434 (36.2%) had a higher education and 340 (28.3%) worked in business. The sex distribution of the last child was similar: 49.0% (588) males and 51.0% (612) females. Most of the women lived in rural areas (78.7%; 944).

**Table 1:** Characteristics of the respondents, Bangladesh, 2023

Characteristic	No. (%) (*n* = 1200)
**Mother’s age at birth of the last child, in years**	
≤ 19	123 (10.2)
20–34	962 (80.2)
≥ 35	115 (9.6)
**Mother’s education**	
No formal education	115 (9.6)
Primary school	233 (19.4)
Secondary school	480 (40.0)
Higher education	372 (31.0)
**Mother in formal employment**	
Yes	372 (31.0)
No	828 (69.0)
**Partner’s education status**	
No formal education	180 (15.0)
Primary school	211 (17.6)
Secondary school	375 (31.2)
Higher education	434 (36.2)
**Partner’s occupation**	
Agriculture worker	183 (15.3)
Labourer	262 (21.8)
Office worker	335 (27.9)
Self-employed businessman	340 (28.3)
Other	80 (6.7)
**Sex of the last child**	
Male	588 (49.0)
Female	612 (51.0)
**Number of children ever born**	
1–2	892 (74.3)
> 2	308 (25.7)
**Household wealth status**	
Poorest	238 (19.8)
Poor	240 (20.0)
Middle	243 (20.3)
Richer	237 (19.8)
Richest	242 (20.2)
**Place of residence**	
Urban	256 (21.3)
Rural	944 (78.7)
**District**	
Kurigram	288 (24.0)
Mymensingh	296 (24.7)
Pabna	306 (25.5)
Satkhira	310 (25.8)

**Fig. 2:**
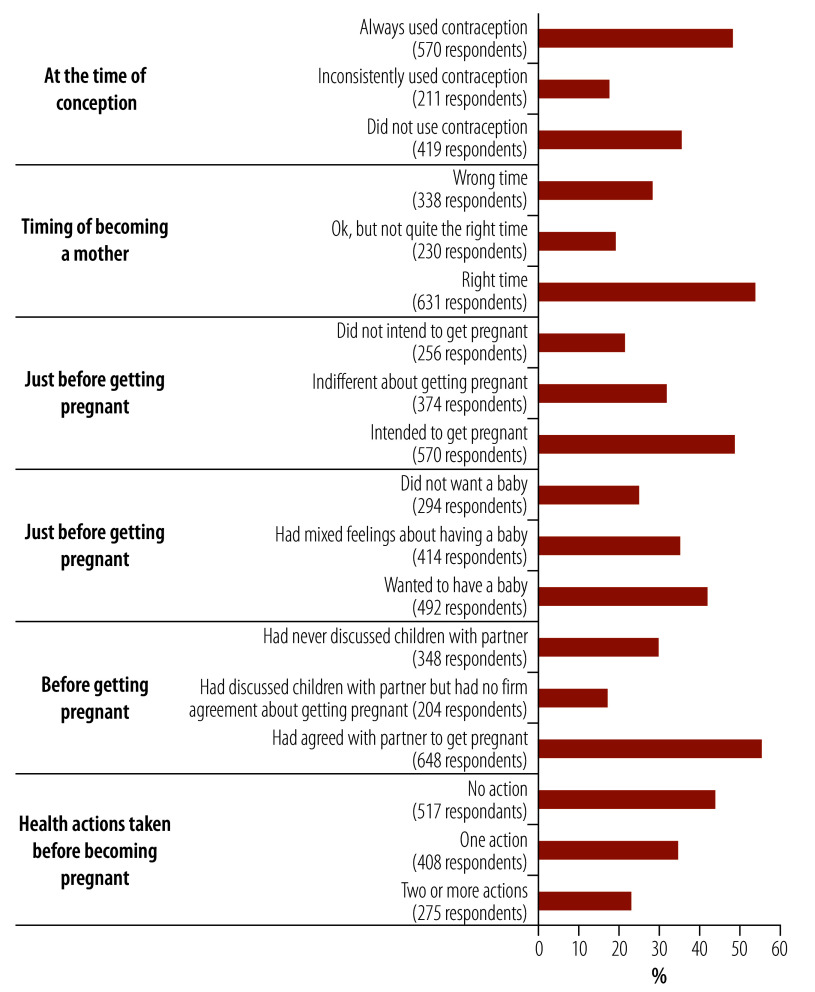
Women’s response to questions on the London Measure of Unintended Pregnancy, Bangladesh, 2023

The prevalence of unplanned pregnancy was higher with the London Measure of Unplanned Pregnancy (31.0%; 373/1200) compared with the DHS measure (24.3%; 292/1200; online repository).^17^ Based on the London measure, 47.5% (570) of the respondents intended to become pregnant at conception, and 52.6% (631) reported that their pregnancy occurred at the right time. Additionally, 54.0% (648/1200) of pregnancies occurring with mutual agreement between spouses. In contrast, 29.0% (348) of women reported that they never discussed pregnancy with their partner. Before becoming pregnant 43.1% (517) reported that they had not taken any health actions, such as taking folic acid or seeking medical or health advice (Fig. 2). A total of 27.1% (325) of responses to pregnancy intention were discordant between the DHS measure and the London Measure of Unplanned Pregnancy (Table 2).

**Table 2:** Comparison of pregnancy intention assessed by the DHS and the London Measure of Unplanned Pregnancy, Bangladesh, 2023

London measure^a^	DHS measure, no.		Total, no. (%)
	Unintended	Intended	
Unplanned	194^b^	179^c^	373 (31.1)
Ambivalent	30^c^	48^c^	78 (6.5)
Planned	68^c^	681^b^	749 (62.4)
**Total, no. (%)**	**292 (24.3)**	**908 (75.7)**	**1200 (100.0)**

DHS: Demographic and Health Survey.

^a^ Classified based on scores where: 0–3: unplanned pregnancy; 4–9: ambivalent about pregnancy; and 10–12: planned pregnancy (online repository).^17^

^b^ Concordant responses in both measures.

^c^ Discordant responses between the two measures.

Note: total concordant responses were 875 (72.9%); total discordant responses were 325 (27.1%).

Several factors associated with discordant responses on pregnancy intention: older respondent age, female sex of the last child, having more than two children, belonging to the poor wealth quintiles, living in rural areas and living in Kurigram district were associated with a higher likelihood of discordant responses in the crude analysis. Secondary and tertiary education of the mother and being in the richest wealth quintile were significantly associated with lower likelihood of discordance (Table 3). 

**Table 3:** Factors associated with discordant responses in pregnancy intention measured by the DHS and London Measure of Unplanned Pregnancy, Bangladesh, 2023

Variable	Discordant response	
	Crude OR (95% CI)	Adjusted OR (95% CI)
**Mother’s age at birth of the last child, in years **		
≤ 19	1.00	1.00
20–34	1.28 (1.10–1.49)	1.32 (1.06–1.53)
≥ 35	1.45 (1.08–1.92)	1.38 (1.06–1.88)
**Mother's education**		
No formal education	1.00	1.00
Primary school	0.78 (0.63–1.26)	0.82 (0.67–1.42)
Secondary school	0.94 (0.82–0.99)	0.96 (0.88–0.99)
Higher education	0.82 (0.68–0.96)	0.84 (0.63–0.95)
**Mother’s formal employment **		
Yes	1.00	1.00
No	1.14 (0.96–1.32)	1.15 (1.01–1.38)
**Partner’s education status**		
No formal education	1.00	1.00
Primary school	0.93 (0.83–1.03)	0.96 (0.85–1.07)
Secondary school	0.91 (0.81–1.04)	0.95 (0.85–1.11)
Higher education	0.86 (0.78–0.94)	0.89 (0.79–0.98)
**Partner’s occupation**		
Labourer	1.00	1.00
Agriculture worker	1.23 (0.93–1.53)	1.26 (0.95–1.56)
Office worker	1.13 (0.80–1.46)	1.18 (0.85–1.48)
Self-employed businessman	1.15 (0.84–1.46)	1.18 (0.82–1.48)
Other	1.06 (0.92–1.20)	1.11 (0.88–1.26)
**Sex of the last child**		
Male	1.00	1.00
Female	1.38 (1.08–1.72)	1.34 (1.06–1.67)
**Number of children ever born **		
1–2	1.00	1.00
> 2	1.24 (1.09–1.39)	1.16 (1.07–1.40)
**Household wealth status**		
Poorest	1.39 (1.05–1.78)	1.32 (1.06–1.82)
Poorer	1.31 (1.02–1.64)	1.34 (1.06–1.76)
Middle	1.00	1.00
Richer	0.95 (0.86–1.04)	0.98 (0.87–1.08)
Richest	0.91 (0.87–0.95)	0.94 (0.87–1.01)
**Place of residence**		
Urban	1.00	1.00
Rural	1.26 (1.04–1.48)	1.30 (1.03–1.51)
**Districts**		
Pabna	1.00	1.00
Mymensingh	0.98 (0.92–1.06)	0.99 (0.93–1.05)
Satkhira	1.00 (0.99–1.03)	1.03 (0.98–1.05)
Kurigram	1.06 (1.01–1.10)	1.08 (1.02–1.17)

CI: confidence intervals; DHS: Demographic and Health Survey; OR: odds ratio.

These findings remained mostly consistent in the adjusted model. Mothers aged 20–34 and ≥ 35 years had 32% (95% CI: 1.06–1.53) and 38% (95% CI: 1.06–1.88) increased odds of discordant responses in pregnancy intention, respectively, compared with mothers aged ≤ 19 years. Mothers with secondary (adjusted OR: 0.96; 95% CI: 0.88–0.99) and higher (aOR: 0.84; 95% CI: 0.63–0.95) education had decreased odds of discordant responses compared with women with no formal education or primary-school education. Mothers with no formal employment had greater odds of discordant responses (aOR: 1.15; 95% CI: 1.01–1.38) than employed mothers. Mothers whose partners had a higher education had lower odds of discordant responses than mothers with partners without formal education (aOR: 0.89; 95% CI: 0.79–0.98). Women with more than two children had higher odds of discordant responses compared to those with one to two children (aOR: 1.16; 95% CI: 1.07–1.40). Mothers in the poorest (aOR: 1.32; 95% CI: 1.06–1.82) and poorer (aOR: 1.34; 95% CI: 1.06–1.76) quintiles were more likely to report discordant responses than mothers in the middle wealth quintile. Rural mothers were 30% (aOR: 1.30; 95% CI: 1.03–1.51) more likely to report discordant responses than urban mothers. Lastly, mothers in Kurigram district had increased odds of discordant responses (aOR: 1.08; 95% CI: 1.02–1.17) compared with mothers in Pabna district (Table 3).

## Discussion

We found a higher prevalence of unplanned pregnancy reported through the London Measure of Unplanned Pregnancy compared with the DHS measure. Additionally, more than a quarter of women provided discordant responses about pregnancy intention between the two measures. Predictors of discordant responses included older age; female sex of the last child; having more than two children; not being in formal employment; belonging to the poor wealth quintiles; living in a rural area; and living in the Kurigram district. These findings suggest the actual occurrence of unintended pregnancy in Bangladesh may exceed the current DHS estimate of 25.2%.^3^ Therefore, the burden on maternal and child health in other low- and middle-income countries related to unintended pregnancy may also be underestimated if based on DHS estimates.

The reported prevalence of unintended pregnancy measured through the DHS method in our study aligns with national estimates in Bangladesh and other low- and middle-income countries.^1^^,^^3^^,^^18^^,^^19^ However, using the more comprehensive London Measure of Unplanned Pregnancy showed a considerably higher prevalence of unplanned pregnancy, with 37.6% (451/1200) of pregnancies classified as either unplanned or ambivalent compared with the DHS estimate of 24.3%. The London Measure of Unplanned Pregnancy gave a more nuanced understanding of unintended pregnancy, with only half of pregnancies occurring with mutual agreement between spouses. Additionally, almost half of women reported not taking any health actions before pregnancy, indicating inadequate counselling on family planning and health check-ups before pregnancy. These findings cannot be easily validated due to limited data on pregnancy intention measured through the London Measure of Unplanned Pregnancy in Bangladesh and other low- and middle-income countries. Various factors could contribute to the higher estimate of unintended pregnancy using the London Measure of Unplanned Pregnancy. This measure collects feelings about a specific pregnancy from both partners, unlike the DHS measure, which focuses solely on women’s feelings. Moreover, the London measure assesses pregnancy timing through multiple questions, while the DHS measure asks a single question about feelings at conception. Consequently, women can recall pregnancy details such as contraception use and failure, as well as partner feelings more accurately. Despite discrepancies in the results of the two measures, both approaches report high rates of unintended pregnancy. These high rates emphasize the need for comprehensive family planning services that address social norms and misconceptions around pregnancy, and provide counselling on family planning and preconception care.^11^^,^^12^ Neglecting these issues can lead to adverse maternal and child health outcomes.

Our findings highlight common mixed feelings or uncertainty about pregnancy intention among women in Bangladesh, with one in four responses on pregnancy intention being discordant. The responses of younger women and those with two or more children were more likely to be discordant in relation to pregnancy intention between the two measures, possibly due to desires to delay or limit childbirth.^3^^,^^20^^–^^22^ Additionally, the higher likelihood of discordance for women whose last child was female may reflect the persistent preference for male children in Bangladesh.^23^ Women may have wanted this child but reported it as unintended when it was a female child. The use of the two approaches to measure pregnancy intention may have led to these differences being captured, resulting in discordant responses. The preference for a son is particularly prevalent among women from lower wealth quintiles and rural areas, who rely heavily on agriculture and view male children as primary workers and caregivers. This preference is also linked to concerns about continuation of family lineage and name preservation.^23^^–^^27^ However, education played an important role in reducing discordance in pregnancy intention, as educated parents were less likely to prefer a specific sex for their child and had better access to family planning and contraception, granting them greater control over pregnancy planning.^27^^–^^29^

The high prevalence of discordance in rating pregnancy intention between the two measures suggests that many women have conflicting feelings about their pregnancies, which can adversely affect various aspects of their lives. For instance, women with contradictory feelings may delay or have limited access to maternal health-care services, as indicated by previous research.^3^^,^^6^^,^^7^^,^^30^ Additionally, women with unintended pregnancies or pregnancy ambivalence are more likely to engage in harmful behaviours during pregnancy, such as smoking, and have higher rates of depression and anxiety, often stemming from concerns about their career or education.^4^^,^^31^ These factors collectively contribute to increased risks of adverse maternal and child health outcomes, including the already high rates of maternal and child mortality observed in Bangladesh and other low- and middle-income countries.^5^^,^^8^

A strength of our study is the large sample, which exceeded the calculated size and enhanced the statistical power of the study. Additionally, using both the DHS measure and the London Measure of Unplanned Pregnancy offers a greater understanding of the extent of unintended pregnancy. However, our study has some limitations. The cross-sectional design of the study does not allow causal inferences. In addition, the inclusion criteria restricted the sample to women accessing postnatal health-care services at selected hospitals and hence excluded women who did not visit health-care facilities for such services, thus limiting the generalizability of the findings. Nevertheless, given the increasing accessibility of postnatal care,^5^^,^^7^ the impact of this limitation is likely minimal. The use of self-reported interviews may also introduce reporting bias into the data, as some women may have been susceptible to providing socially desirable responses. The women, however, were assured of the anonymity of their responses before the interviews, which were carried out in a separate room with only the woman and the interviewer. During data collection, questions were asked carefully, with examples given for important ones. Therefore, any social desirability biases were likely to be random. Lastly, data on factors such as social norms about child sex preference were not collected because of the lack of relevant data in the survey, although they potentially influence discrepancies between the measures. 

Our findings have important policy implications for Bangladesh. The high prevalence of unintended pregnancy, coupled with substantial discordance between the applied measures, underscores the significant burden of unintended pregnancy and its underreporting at a community level. The low uptake of maternal health-care services in instances of unintended pregnancy suggests an elevated risk of adverse maternal and child health outcomes, including maternal and child mortality.^4^^–^^7^ Consequently, action is needed to implement comprehensive family planning services and encourage the uptake of contraception, with a focus on younger, economically disadvantaged and less educated women. These efforts should be integrated into existing family planning services and scaled up throughout Bangladesh. Surveys in low- and middle-income countries, including Bangladesh, should incorporate updated measures of unintended pregnancy, such as the London Measure of Unplanned Pregnancy, into their questionnaires (the DHS measure) to enable more accurate monitoring of the prevalence of unintended pregnancy. This inclusion would not incur additional expenses given the large number of variables already incorporated in the DHS surveys. The more accurate information could help guide programmes aimed at reducing the occurrence of unintended pregnancy and associated adverse outcomes.
