# Corrigendum

**DOI:** 10.2471/BLT.26.100326

**Published:** 2026-03-01

In: Khan NMd, Khanam SJ, Harris ML. Comparison of two survey methods for estimating unplanned pregnancy, Bangladesh. Bull World Health Organ. 2024 Aug 1;102(8):562–70.

On page 562, the fourth sentence in the third paragraph should read:

 “Furthermore, the extent of discordance between the two measures and its contributing factors remain largely unreported.”

On page 563, the first sentence under Ethical considerations should read:

“The Human Research Ethics Committee of the Asian Institute for Disability and Development approved the final version of the questionnaire and the data collection method (approval number: Southasia-hrec-2022-11-02)”

